# *Barnesiella intestinihominis* improves gut microbiota disruption and intestinal barrier integrity in mice with impaired glucose regulation

**DOI:** 10.3389/fphar.2025.1635579

**Published:** 2025-10-08

**Authors:** Xiaojia Liu, Ling Wang, Bing Huang, Yi Jiao, Yaqun Guan, Rebiya Nuli

**Affiliations:** ^1^ Department of Human Parasitology, School of Basic Medical Sciences, Xinjiang Medical University, Urumqi, Xinjiang, China; ^2^ Department of Biochemistry and Molecular Biology, School of Basic Medical Sciences, Xinjiang Medical University, Urumqi, Xinjiang, China; ^3^ Xinjiang Key Laboratory of Molecular Biology for Endemic Disease, Xinjiang Medical University, Urumqi, Xinjiang, China

**Keywords:** *Barnesiella intestinihominis*, gut microbiota, impaired glucose regulation, intestinal barrier, insulin resistance

## Abstract

**Introduction:**

Impaired glucose regulation (IGR) is a prediabetic state closely associated with gut microbiota dysbiosis. Our previous metagenomic analysis identified a significant deficiency of *Barnesiella intestinihominis* (*B. intestinihominis*) in IGR patients (*p* < 0.01). The present study was therefore designed to investigate the therapeutic potential of *B. intestinihominis* supplementation in a high-fat diet (HFD)-induced IGR mouse model and to explore its potential mechanisms of action.

**Methods:**

A mouse model of IGR was established by HFD. The treatment group received a daily supplementation of live *B. intestinihominis* (1×10^8^ CFU) for 5 weeks. Gut microbiota composition was analyzed. Colonic expression levels of tight junction proteins (ZO-1 and occludin) and cytokines (IL-10, TNF-α, IL-6) were measured. *In vitro* experiments using Caco-2 human intestinal epithelial cells were conducted to assess the direct effects of *B. intestinihominis*. *B. intestinihominis* fermentation broth, heat-inactivated bacterial solution, and bacterial solution were co-cultured with Caco-2 cells. Cell viability was assessed using the CCK-8 assay, and the expression levels of tight junction proteins were evaluated. Trans-epithelial electrical resistance (TEER) and alkaline phosphatase activity were also assessed in the Caco-2 model.

**Results:**

Daily supplementation with *B.intestinihominis* significantly attenuated HFD-induced hyperglycemia in mice. It also modulated the gut microbiota, evidenced by an increased abundance of beneficial *Ligilactobacillus* and a reduction in pathogenic *Lachnoclostridium*. Furthermore, *B. intestinihominis* administration upregulated the expression of colonic tight junction proteins (ZO-1 and occludin) and the anti-inflammatory cytokine IL-10, while simultaneously inhibiting the pro-inflammatory mediators TNF-α and IL-6. *In vitro*, the fermentation broth of B.intestinihominis (10%) increased Caco-2 cell viability, and heat-inactivated bacteria (1×10^7^ CFU) enhanced ZO-1 expression. However, neither treatment significantly affected trans-epithelial electrical resistance (TEER) nor alkaline phosphatase activity in Caco-2 cells.

**Discussion:**

These findings suggest that intestinal probiotics *B. intestinihominis* may ameliorate IGR by modulating the gut microbiota, enhancing intestinal barrier integrity, and attenuating inflammation, thus supporting their potential as a therapeutic intervention for metabolic disorders.

## 1 Introduction

The progression from normoglycemia to type 2 diabetes mellitus (T2DM) typically occurs through intermediate metabolic states collectively termed impaired glucose regulation (IGR). According to the 10th edition of the International Diabetes Federation (IDF) Diabetes Atlas, the global prevalence of IGR reached 860 million cases in 2021, with projections indicating a 15.4% increase to 992 million by 2030. Individuals in IGR are at an elevated risk of developing T2DM compared to those with normal blood glucose levels, with annual progression rates ranging from 3.0% to 11.0% (Ma et al., 2019). While lifestyle interventions remain cornerstone management strategies, persistent hyperglycemia in these patients substantially increases cardiovascular complication risks, making IGR and T2DM prevention a critical public health priority (Tian et al., 2020; Xie et al., 2021).

Gut microbiota (GM) dysbiosis and persistent inflammatory responses are hallmarks of type 2 diabetes mellitus (T2DM). T2DM is associated with increased intestinal permeability, which leads to disruption of the intestinal barrier and induces metabolic endotoxemia and metabolic inflammation (Everard et al., 2013). This understanding has spurred investigation into GM modulation strategies, particularly through targeted bacterial transplantation. Current evidence demonstrates that specific probiotic strains, including *Lactobacillus plantarum HAC01* and *Lactobacillus plantarum Y15*, can significantly improve metabolic parameters in T2DM by multiple mechanisms: suppressing hepatic gluconeogenesis, enhancing pancreatic β-cell function, and improving overall glucose homeostasis (Lee et al., 2021; Liu et al., 2022). Similarly, *Lactobacillus paracasei HII01* (*LPPH*) shows promise in remodeling GM composition, reducing pro-inflammatory TNF-α expression in skeletal muscle, and enhancing glucose utilization through AKT phosphorylation and GLUT4 translocation (Toejing et al., 2020). These findings collectively support the therapeutic potential of beneficial bacterial supplementation in metabolic disorders. Our previous clinical investigations identified significant depletion of *Barnesiella spp*. in both IGR and T2DM patients (Nuli et al., 2019; Xue et al., 2023). Existing literature documents *Barnesiella*’s capacity to inhibit colonization by vancomycin-resistant *Enterococcus faecalis* (Ubeda et al., 2013), its negative correlation with diarrheal incidence in calves (Fan et al., 2021), and the specific strain *B. intestinihominis*’ ability to potentiate cyclophosphamide’s antitumor effects (Daillère et al., 2016). This gram-negative, anaerobic commensal bacteria, originally isolated from healthy human feces, exhibits glucose-metabolizing capabilities producing succinate and acetate as major end products (Moroto et al., 2008), positioning it as a potentially significant short-chain fatty acids (SCFAs) producer in gut homeostasis.

However, it remains unclear whether *B. intestinihominis* is associated with the intestinal barrier in maintaining GM homeostasis in IGR and few reports on the role of *B. intestinihominis* in the diabetes. We used a gut bacterial transplantation approach to intervene in an high-fat diet (HFD) induced IGR mice model and Caco-2 cell model of impaired intestinal barrier to study the potential effects of *B. intestinihominis* on GM and disease progression in IGR mice and its impact on the gut barrier.

## 2 Materials and methods

### 2.1 Animals and ethical approval

Forty-two male C57BL/6J mice (SPF grade, 5 weeks old, 18.0–20.0 g) were obtained from Hunan Slaughter Kingda Laboratory Animal Co. Ltd. (Changsha, Hunan, China) and maintained in specific pathogen-free (SPF) conditions with controlled temperature (22 °C ± 3 °C), humidity (50% ± 5%), and 12 h light/dark cycles. The experimental protocol was approved by the Ethics Review Committee of Xinjiang Medical University (Urumqi, China) (Approval number: IACUC-20220725-37), with all procedures conducted in strict accordance with the “Guide for the Care and Use of Animals” following National Institute of Health guidelines for animal welfare.

### 2.2 Culture and preparation of *B. intestinihominis*

*Barnesiella intestinihominis (B. intestinihominis*, strain: DSM 21032) was obtained from the German Microbial Strain Conservation Center DSMZ and cultured in minced meat broth (Mingzhou, Zhejiang, China, K0747) under anaerobic conditions (10% CO_2_, 10% H_2_, 80% N_2_). A 1% inoculum was transferred to fresh broth and cultured to the late logarithmic growth phase. The bacterial cells were harvested by centrifugation (12,000 rpm, 4 °C, 10 min) to achieve a final concentration of 1 × 10^8^ CFU/mL for animal experiments and 1 × 10^7^ CFU/mL for *in vitro* cell experiments. For fermentation medium preparation, centrifuge the bacterial suspension (12,000 rpm, 15 min, 4 °C), filter the supernatant (0.22 μm filter membrane), and store at −20 °C. Thermally inactivated bacteria were collected by centrifugation (12,000 rpm, 4 °C, 10 min), washed three times with PBS (pH 7.0 ± 0.2), resuspended in sterile water to a concentration of 1 × 10^7^ CFU/mL, and prepared by autoclaving at 121 °C for 20 min.

### 2.3 Induction of experimental IGR

The establishment of a impaired glucose regulation (IGR) model was carried out as described by our previous study (Zhao et al., 2022). IGR was induced in C57BL/6J mice via 8-week 45% high-fat diet (HFD) (Medicience, Jiangsu, China, MD12032) feeding. Assess glucose metabolism and insulin sensitivity in mice using the Intraperitoneal Glucose Tolerance Test (IPGTT) and Intraperitoneal Insulin Tolerance Test (IPITT). Mice with fasting plasma glucose (FPG) > 5.6 mmol/L or 2-h glucose tolerance test (2h-PG) > 7.8 mmol/L were diagnosed with IGR and divided into the IGR group and the *B. intestinihominis*-treated group. Based on previous research results, *B. intestinihominis* bacterial culture solutions at concentrations of 1 × 10^7^ CFU/mL, 0.5 × 10^8^ CFU/mL, and 1 × 10^8^ CFU/mL did not exhibit toxic effects on mice (Wang et al., 2024). Preliminary studies revealed that when C57BL/6J mice were supplemented with 1 × 10^8^/mL *B. intestinihominis*, the DNA copy number of this strain in feces significantly increased, demonstrating optimal colonization efficacy. The *B*. *intestinihominis*-treated group received daily 0.2 mL (1 × 10^8^ CFU) *B. intestinihominis* for 5 weeks, while controls received culture medium. IGR mice were maintain with HFD feeding for 5 weeks, and other mice were fed with normal chow diet. Weekly body weight and fasting blood glucose measurements were conducted post-gavage. For IPGTT, 16-h fasted mice received 1 g/kg 20% glucose (tail vein sampling), with blood glucose monitored using Roche AUUC-CHEK Performa (Roche Diabetes Care GmbH, Mannheim, Germany). Mice were anesthetized by intraperitoneal injection of 2% concentration of sodium pentobarbital at a dose of 0.1 mL/50 g. After anesthesia, blood was collected by removing the eyeballs, and the mice were executed by cervical dislocation. Collections included feces samples and colon tissues, with partial tissue fixed in 4% paraformaldehyde (Bioshap, China, BL539A) and remaining tissue snap-frozen at −80 °C.

### 2.4 Enzyme-linked immunosorbent assay (ELISA)

The kit was used to measure the concentrations of insulin (Proteintech, Shanghai, China, KE10089), and lipopolysaccharide (LPS) in mouse serum (Zcibio, Shanghai, China, ZC-39007), and interleukin-6 (IL-6) (Zcibio, Shanghai, China, ZC-37988), Interleukin-10 (IL-10) (Zcibio, Shanghai, China, ZC-37962), and tumor necrosis factor-α (TNF-α) (Zcibio, Shanghai, China, ZC-39024) in colonic tissues, and all kits were performed according to the manufacturer’s instructions.

### 2.5 HE staining

After euthanasia, the colonic tissues of mice were fixed in 4% paraformaldehyde (Bioshap, China, BL539A). Paraformaldehyde-fixed tissue slides were stained with HE staining. All processes were performed according to standard protocols followed by microscopy examination. The entire cross section was scanned and imaged in a digital pathology system (Leica Aperio LV1).

### 2.6 GM 16S rRNA sequencing

GM bacterial DNA was extracted from fecal samples using a bacterial gDNA stool extraction kit (Qiagen, Hilden, Germany) and the DNA quality was assessed using 1% agarose gel electrophoresis. The *16S rRNA* region V3–V4 was amplified using barcoded universal primers (forward 338F: 5′-ACT​CCT​ACG​GAG​GCA​GCA​G-3′; reverse 806R: 5′-GGACTACHVGGTWTCTAAT-3′). GM *16S rRNA* sequencing (Shanghai Majorbio Bio-Pharm Technology, Shanghai, China) and data analyses were performed as described (Zhao et al., 2022). The analysis of alpha diversity involved calculating the Chao and Shannon indices based on observed operational taxonomic units. The partial least squares discriminant analysis (PLS-DA) and non-metric multidimensional scaling (NMDS) were used for beta diversity analysis. The LEfSe method was applied to identify differences in abundance.

### 2.7 Caco-2 cell monolayer barrier transmembrane resistance (TEER) assay and alkaline phosphatase activity measurement

Caco-2 cells were human colorectal adenocarcinoma cells purchased from the Cell Resource Center, Institute of Basic Medical Sciences, Chinese Academy of Medical Sciences. The cells were cultured in DMEM medium (Procell, Wuhan, China) containing 20% fetal bovine serum (Life-ilab, Shanghai, China, AC03L0555) and 1% penicillin-streptomycin double antibody (GE-HYCLONE, SV30010) at 37 °C with 5% CO_2_.

Caco-2 cells in logarithmic growth phase were seeded on Transwell inserts (0.4 μm pore, 0.33 cm^2^) at 2 × 10^4^ cells/well. Medium was changed every other day initially, then daily after 1 week, forming monolayers by day 21. Mature monolayers were treated with 100 μg/L TNF-α for 4 h, washed with PBS, then exposed to either 10% *B. intestinihominis* fermentation broth or 10^7^ CFU/mL live bacteria for 6 h. The transmembrane electrical resistance (TEER) was measured for the monolayer barrier of Caco-2 cells. The TEER value calculated according to the following formula: TEER = (TEER_T_–TEER_C_) × A, where TEER_T_ represents the TEER value (Ω) of the measured transwell chamber with cells, TEER_C_ is the TEER value (Ω) of the transwell chamber in the blank group, and A is the membrane area of the transwell chamber (0.33 cm^2^).

For alkaline phosphatase (ALP) activity, differentiated Caco-2 monolayers were treated and analyzed using a commercial ALP kit (Beyotime, P0321S) following manufacturer’s protocol.

### 2.8 Effects of *Barnesiella intestinihominis* on Caco-2 cells *in vitro*

Cells growing in logarithmic phase were inoculated in the inner lumen of a six-well plate (BIOFIL, China, TCP010006) with cell density up to 80%, and Caco-2 cells were intervened using 2.5% Dextran Sulfate Sodium (DSS) for 48 h, washed twice with PBS after 24 h, and finally treated with 10% *B. intestinihominis* fermentation broth, 10^7^ CFU/mL *B. intestinihominis* heat-inactivated bacterial solution, and *B. intestinihominis* bacterial solution. The mRNA and protein expression level of ZO-1 and occludin measured by RT-qPCR and Western Blot in Caco-2 cell.

### 2.9 mRNA extraction and RT-qPCR

Total RNA was extracted from mouse colon tissues using TRIzol reagent (Invitrogen, Life Technology, California, United States) and from Caco-2 cells using RNA extraction solution (Servicebio, Wuhan, China, G3013), with concentration/purity assessed by spectrophotometry. cDNA was synthesized from 500 ng RNA using Prime Script RT kit (Takara, Kusatsu, Japan). RT-qPCR was performed on ABI 7900HT system (Applied Biosystems Instruments, Thermo Fisher Scientific, Carlsbad, United States) with SYBR Green kit (Qiagen) in 10 μL reactions containing 1 μL cDNA, 5 μL 2× PerfectStart^®^ Green RT-qPCR SuperMix, 0.2 μL passive fluorescent dye (50×), and 0.2 μL each primer (Sangon Biotech, Shanghai, China). Cycling conditions: 94 °C/30°s; 40 cycles of 94 °C/5°s and 60 °C/30°s; melt curve analysis (95 °C/15°s, 60 °C/60°s, 95 °C/1 s). Data were normalized to β-actin and analyzed by 2^−ΔΔCT^ method (Supplementary Material).

### 2.10 Western blot analysis

Total protein was extracted from mouse colon tissues and Caco-2 cells using RIPA lysis buffer (Solarbio, Beijing, China, Cat#R0010), quantified by Bicinchoninic Acid Assay (ThermoFisher, United States, A55860), and denatured with SDS (12.5 μg protein/lane). Proteins were separated by SDS-PAGE, transferred to NC membranes, and blocked with 5% skim milk for 2 h. Membranes were incubated overnight with primary antibodies (ZO-1, occludin, HSP90; Proteintech, Wuhan, China; 1:5,000), followed by HRP-conjugated secondary antibody (Proteintech, Beijing, China; 1:5,000) for 1 h. Target protein expression was visualized using enhanced chemiluminescence.

### 2.11 *In vitro* Caco-2 intestinal epithelial cell intercellular junction immunostaining

To evaluate intercellular junction integrity of the Caco-2 monolayer, Caco-2 cells were separately seeded on to 24-well plates. The Caco-2 cells were intervened using 2.5% DSS for 48 h, washed twice with PBS after 24 h, and finally treated with 10% *B. intestinihominis* fermentation broth, 10^7^ CFU/mL *B. intestinihominis* heat-inactivated bacterial solution, and *B. intestinihominis* bacterial solution. The cell culture supernatant was discarded, and the cells were washed twice with PBS. Cells were fixed using 4% paraformaldehyde (PFA) for 10 min. After washing, 1 mL of 0.3% TritonX-100 was added and the samples were incubated at room temperature for 10 min. Block with 1 mL of 3% bovine serum albumin (BSA) at room temperature for 1 h, then add ZO-1 (Proteintech, Beijing, China; 1:1,000) and occludin (Proteintech, Beijing, China; 1:2,000) primary antibodies were added and incubated overnight at 4 °C. 70 μL of fluorescent secondary antibody against rabbit IgG (H + L) (Cell Signaling Technology, Boston, United States) was added and incubated at room temperature for 1 h, and then photographed using a an inverted microscope (Lecia, Vizsla, Germany).

### 2.12 Statistical analyses

Statistical analysis was performed using GraphPad Prism 10.0. Data are presented as mean ± SD. Two-group comparisons used t-tests; one-way analysis of variance (ANOVA) followed by Bonferroni’s multiple comparisons was performed to compare multiple groups. Biomarker analysis employed LDA effect size. Correlations used Spearman’s R. *P* < 0.05 was considered statistically significant. Images were processed with ImageJ and Adobe Photoshop.

## 3 Results

### 3.1 *Barnesiella intestinihominis* improves impaired glucose regulation in mice

**FIGURE 1:**
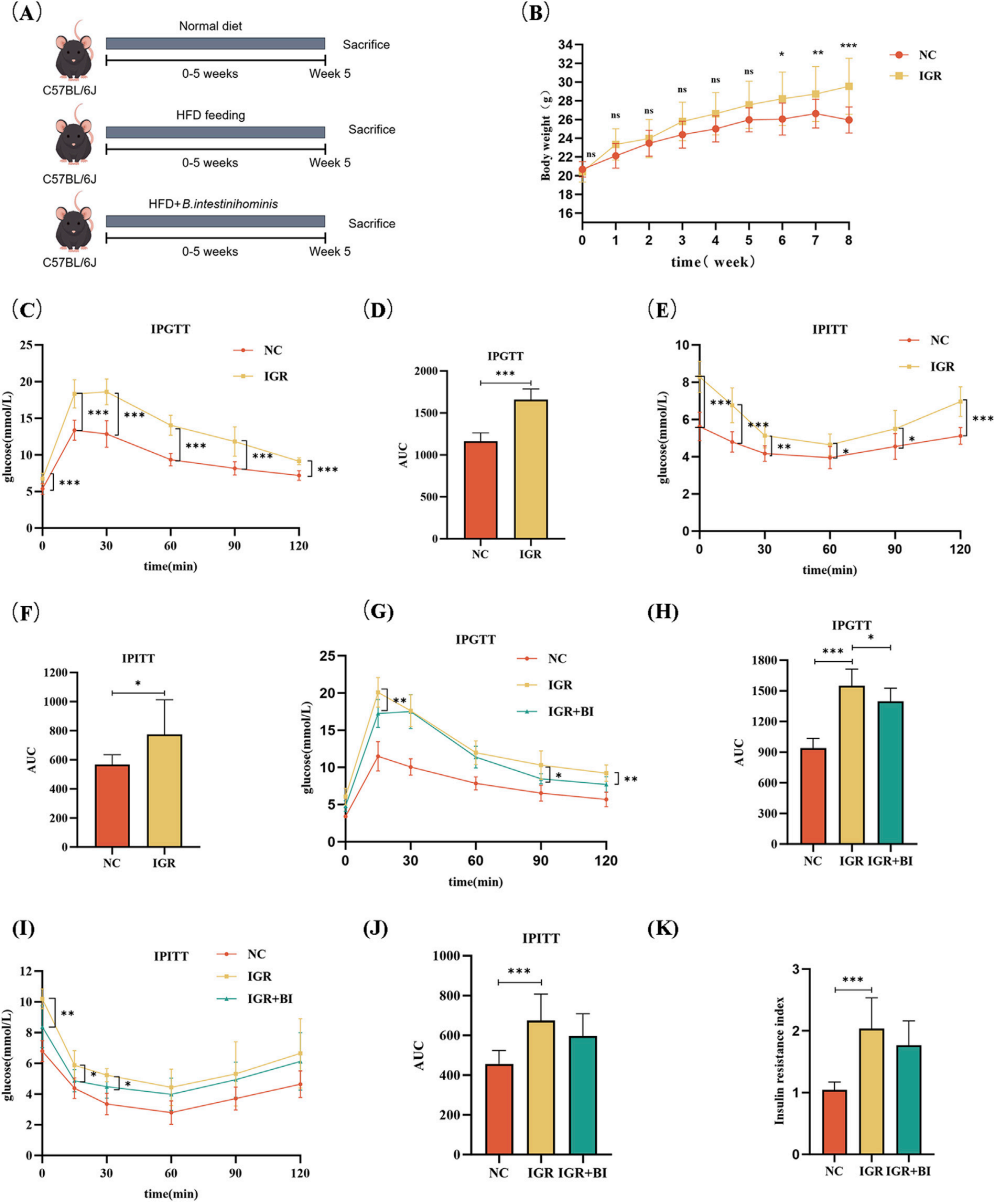
*Barnesiella intestinihominis* improved glucose tolerance in IGR mice with high-fat diet. **(A)** A schematic diagram of the study design. **(B)** Weight changes in IGR mice induced by a high-fat diet during the study period: normal control group (NC group) n = 8, IGR group n = 16. **(C)** IPGTT experimental curve before *B. intestinihominis* treatment, **(D)** is the area under the curve of **(C)**. **(E)** IPITT experimental curve before *B. intestinihominis* treatment, **(F)** is the area under the curve of **(E)**. **(G)** IPGTT experimental curve after *B. intestinihominis* treatment, **(H)** is the area under the curve of **(G)**. **(I)** IPITT experimental curve after *B. intestinihominis* treatment, **(J)** is the area under the curve of **(I)**, n = 8, **(K)** insulin resistance index of mice after *B. intestinihominis* treatment (*p < 0.05, **p < 0.01, ***p < 0.001).

To identify the potential beneficial effect of *B. intestinihominis*, mice with IGR induced by a high-fat diet were treated with live *B. intestinihominis* daily for 5 weeks (Figure 1A). Following HFD induction, the IGR group exhibited significantly higher body weight compared to the normal chow (NC) control group (*p* < 0.01), as demonstrated in Figure 1B. IPGTT and IPITT analyses revealed significantly elevated area under the curve (AUC) values in the IGR group compared to NC controls (*p* < 0.05), confirming successful establishment of the IGR model (Figures 1C–F). Notably, after 5 weeks of *B. intestinihominis* intervention, the *B*. *intestinihominis*-treated group demonstrated significant improvements in glucose homeostasis. The IPGTT analysis showed a marked reduction in AUC values compared to untreated IGR controls (*p* < 0.05), with significant decreases in both peak glucose levels and 2-h postprandial glucose concentrations (*p* < 0.05). Although not statistically significant, the IPITT AUC values showed a decreasing trend, suggesting improved insulin sensitivity (Figures 1G–J). Meanwhile, it was found that HFD also led to an increase in the Homeostasis Model Assessment of Insulin Resistance (HOMA-IR) index in IGR group, and *B. intestinihominis* alleviated the HOMA-IR index (Figure 1K). These findings collectively demonstrate that *B. intestinihominis* intervention exerts beneficial effects on glucose metabolism in IGR mice, potentially through modulation of insulin sensitivity and glucose homeostasis.

### 3.2 *Barnesiella intestinihominis* ameliorates intestinal inflammation in IGR mice

**FIGURE 2:**
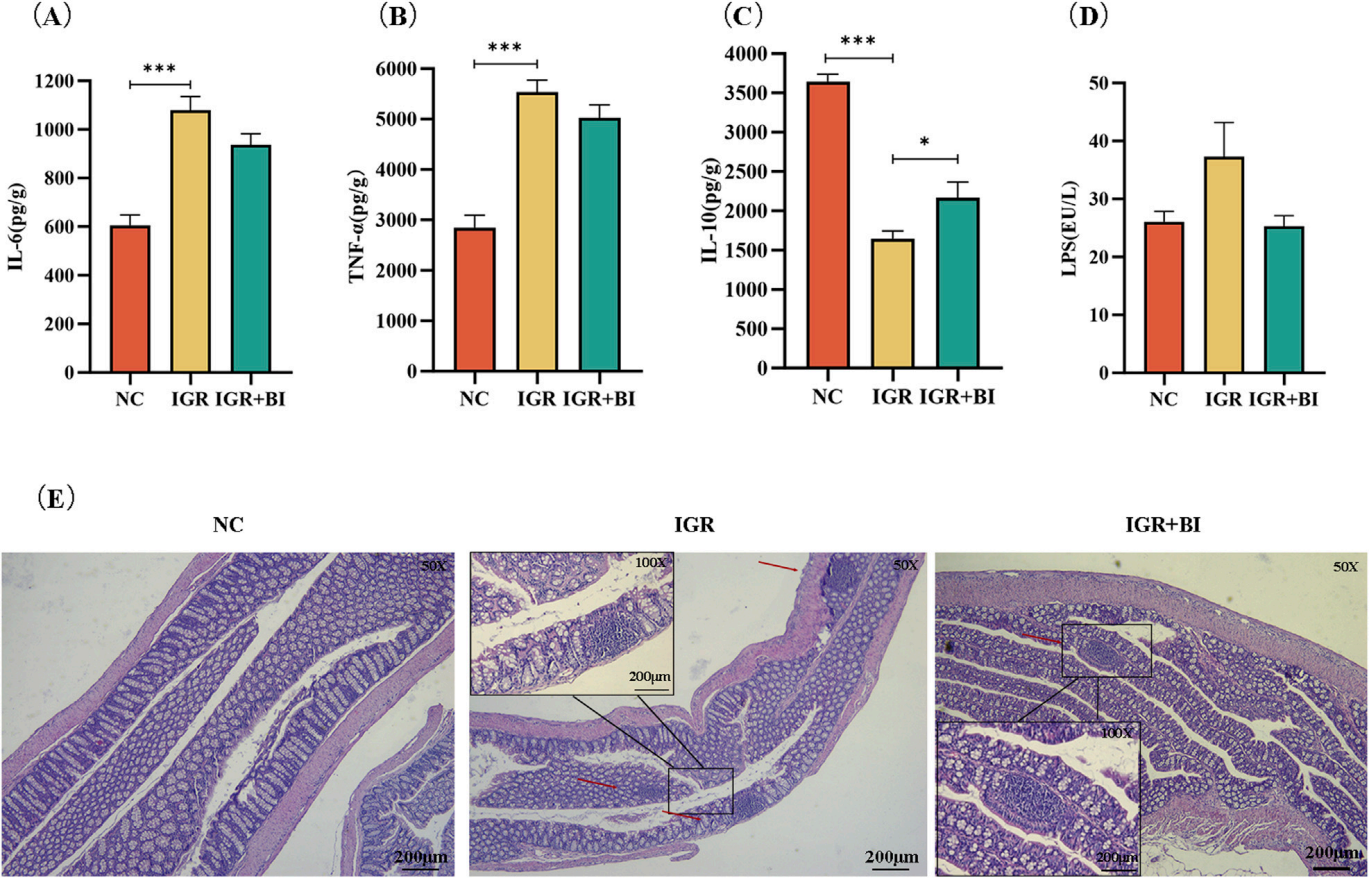
*Barnesiella intestinihominis* improved intestinal inflammation in IGR mice. **(A–C)** Inflammation factors in mouse colon tissue. **(D)** Map of LPS levels in serum of mice. **(E)** HE staining of mouse colon tissue (Small frame magnification: ×50; Large frame magnification: ×200). n = 6, *p < 0.05, **p < 0.01, ***p < 0.001.

To evaluate the anti-inflammatory effects of *B. intestinihominis*, we performed comprehensive analyses of colonic and systemic inflammatory markers. Colonic tissue homogenates and serum samples were collected for quantification of inflammatory cytokines and LPS levels using ELISA. The results revealed significant dysregulation of inflammatory mediators in IGR mice compared to NC controls. Specifically, colonic levels of pro-inflammatory cytokines IL-6 and TNF-α were markedly elevated in the IGR mice (*p* < 0.05), accompanied by a moderate increase in serum LPS levels and significant reduction in the anti-inflammatory cytokine IL-10 (*p* < 0.05). Following *B. intestinihominis* intervention, we observed a significant modulation of the inflammatory profile. Colonic IL-10 levels were substantially increased (*p* < 0.05), while concentrations of pro-inflammatory cytokines (IL-6 and TNF-α) and serum LPS showed a decreasing trend, suggesting restoration of inflammatory homeostasis (Figures 2A–D). Histopathological examination of colon tissues through H&E staining provided further evidence of *B. intestinihominis*-mediated protection. While IGR mice exhibited significant inflammatory cell infiltration compared to NC controls, *B. intestinihominis* treatment markedly attenuated this pathological alteration (Figure 2E). These findings collectively demonstrate that *B. intestinihominis* intervention effectively ameliorates intestinal inflammation in IGR mice through multiple mechanisms, including reduction of pro-inflammatory mediators, enhancement of anti-inflammatory responses.

### 3.3 *Barnesiella intestinihominis* restores high-fat diet induced gut microbiota imbalance

**FIGURE 3:**
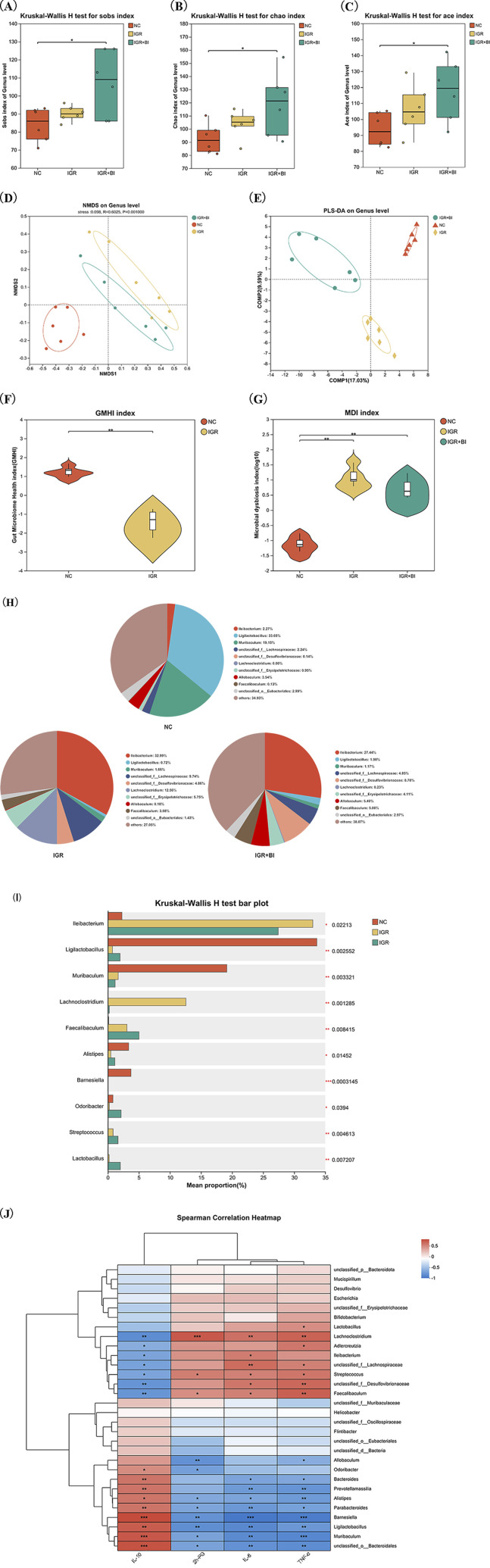
*Barnesiella intestinihominis* restores GM dysbiosis in IGR mice with high-fat diet. **(A–C)** The changes of Sob index, Chao index and Ace indexes **(D)** NMDS map after *B. intestinihominis* intervention. **(E)** PLS-DA map after *B. intestinihominis* intervention. **(F)** GMHI chart of IGR mice with high-fat diet. **(G)** Three sets of MDI maps. **(H)** Pie chart of microbiota composition in three groups of mice. **(I)** Analysis diagram of species composition difference among the three groups of mice. **(J)** Correlation analysis heat map. n = 6, *p < 0.05, **p < 0.01, ***p < 0.001.

To investigate the impact of *B. intestinihominis* on gut microbiota (GM) composition and diversity, we performed 16S rRNA gene sequencing analysis. Alpha diversity assessment, incorporating the Sob, Chao, and Ace indices, revealed no significant differences between the IGR mice and NC groups. However, *B. intestinihominis* intervention significantly enhanced microbial diversity in the *B*. *intestinihominis*-treated group compared to the IGR mice (*p* < 0.05) (Figures 3A–C). The β-diversity analysis using partial least squares discriminant analysis (PLS-DA) and non-metric multidimensional scaling (NMDS) demonstrated distinct clustering patterns among the NC, the IGR mice, and the *B*. *intestinihominis*-treated group, indicating that both HFD feeding and *B. intestinihominis* treatment significantly altered GM composition (Figures 3D,E). Compared with the NC group, the gut microbiota health index (GMHI) of the IGR mice decreased significantly (Figure 3F), and the dysbiosis index (MDI) increased significantly, and the MDI of the mice in the *B*. *intestinihominis*-treated group showed a decreasing trend after the treatment of *B. intestinihominis* (Figure 3G). The GM of the three groups of mice were further analyzed for species composition, and the results of pie charts showed that the richness of the microbiota at genus level of each group were changed. The bacteria with the highest percentage in the NC group were *Ligilactobacillus*, *Muribaculum*, *Allobaculum* and *Ileibacterium*; the highest percentage of genus in the IGR mice were *Ileibacterium*, *Lachnoclostridium*, *Faecalibaculum*, and *Muribaculum*; the genus with the highest percentage in the *B*. *intestinihominis*-treated group were *Ileibacterium*, *Allobaculum* and *Faecalibaculum* (Figure 2H); the results of the analysis of species differentiation of the GM of the three groups of mice showed that the relative abundance *Ligilactobacillus* and *Muribaculum* decreased significantly (*p* < 0.05) in the IGR mice, and the relative abundance of *Ileibacterium* and *Lachnoclostridium* increased significantly (*p* < 0.05); whereas the relative abundance of *Ligilactobacillus* showed an increasing trend in the *B*. *intestinihominis*-treated group after the treatment of *B. intestinihominis*, the relative abundance of *Lachnoclostridium* decreased significantly (*p* < 0.05) (Figure 3I). Further correlation analysis was performed between the GM genus and the expression of inflammatory factors in the colonic tissues and 2h-PG, and the correlation analysis heat map showed that *Ligilactobacillus*, *Muribaculum* and *Barnesiella* showed negative correlation changes with the pro-inflammatory factors IL-6 and TNF-α and the 2h-PG, while positive correlation changes were observed with the anti-inflammatory factor IL-10; in contrast, *Lachnoclostridium* and *Faecalibaculum* showed positively correlated changes with pro-inflammatory factors (IL-6 and TNF-α) and 2h-PG, and negatively correlated changes with the anti-inflammatory factor IL-10 (Figure 3J). These findings collectively demonstrate that *B. intestinihominis* intervention effectively restores HFD-induced GM dysbiosis in IGR mice, promoting a favorable microbial profile associated with improved metabolic and inflammatory status. The observed microbiota modifications suggest that *B. intestinihominis* may exert its beneficial effects through modulation of microbial community structure and function. Taken together, these results indicate that *B. intestinihominis* restores high-fat diet induced GM dysfunction in IGR mice.

### 3.4 *Barnesiella intestinihominis* increases ZO-1 and occludin expression in colon tissue of mice with IGR mice

**FIGURE 4:**
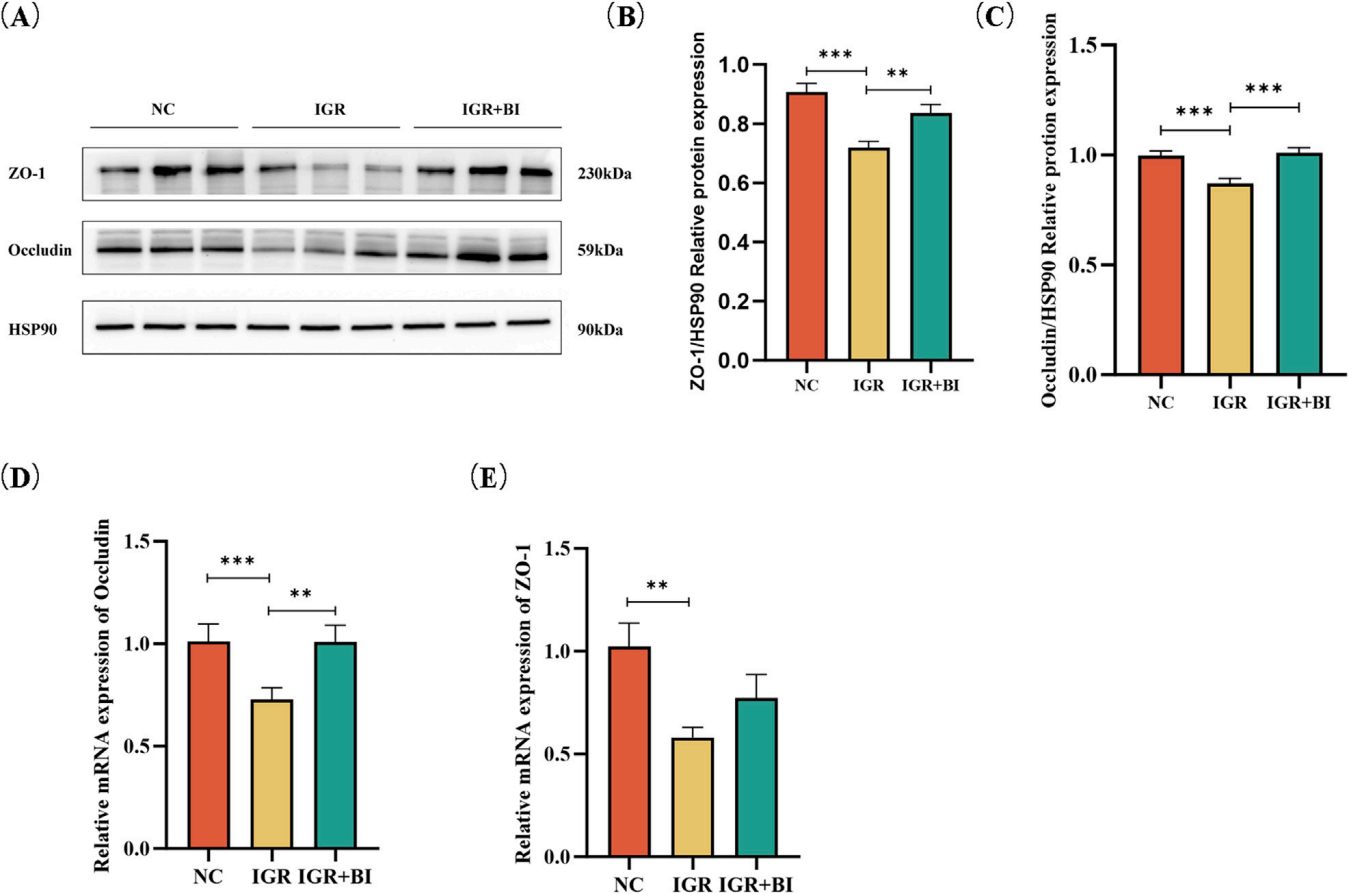
*Barnesiella intestinihominis* intervention increased expression of ZO-1 and occludin in IGR mice. **(A)** Western Blot of ZO-1 and occludin expression in colon tissue. **(B,C)** Quantitative Western Blot of ZO-1 and occludin in colon tissue. **(D,E)** Relative quantitative map of ZO-1 and occludin genes in colon tissue by RT-qPCR. n = 6, *p < 0.05, **p < 0.01, ***p < 0.001.

To evaluate the impact of *B. intestinihominis* on intestinal barrier integrity, we assessed the expression of critical tight junction proteins in colonic tissues. Western blot analysis revealed significant downregulation of ZO-1 and occludin protein expression in the IGR mice compared to NC controls (*p* < 0.05). Notably, *B. intestinihominis* intervention markedly upregulated the expression of both ZO-1 and occludin in the *B. intestinihominis*-treated group compared to untreated the IGR mice (*p* < 0.05) (Figures 4A–C). RT-qPCR detected the mRNA expression levels of ZO-1 and occludin, and the results showed mRNA expression levels ZO-1 and occludin were significantly decreased in the IGR mice compared to the NC group (*p* < 0.05), whereas the mRNA expression level of occludin was significantly increased in the *B*. *intestinihominis*-treated group (*p* < 0.05), and the mRNA expression level of ZO-1 also showed an increasing trend, as shown in Figures 4D,E. In conclusion, *B. intestinihominis* can improve the integrity of the intestinal barrier by increasing the expression of tight junction proteins ZO-1 and occludin in colonic tissues and prevented leaky gut in IGR mice.

### 3.5 *Barnesiella intestinihominis* fermentation broth increases Caco-2 cell viability and heat-inactivated bacterial broth increases ZO-1 expression

**FIGURE 5:**
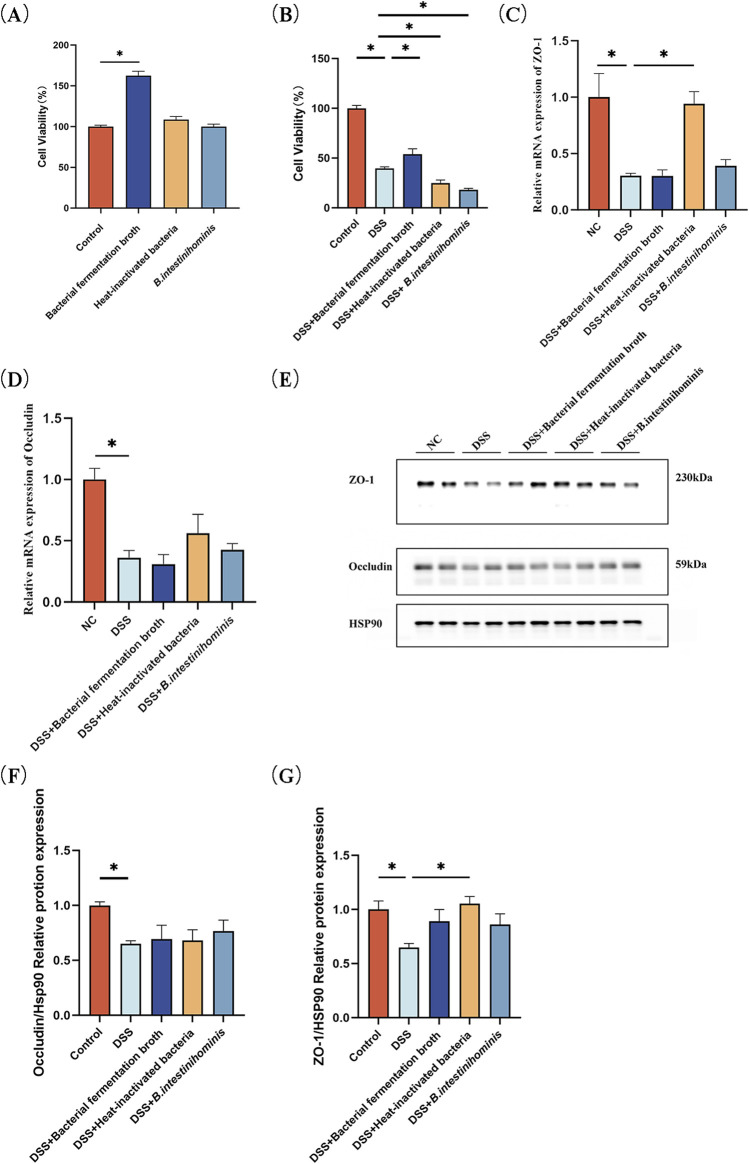
*Barnesiella intestinihominis* fermentation broth increases viability of Caco-2 cell and ZO-1 expression. increased after heat-inactivated broth treatment. **(A)** Effect of bacterial solution on cell viability. **(B)** Effect of co-culture of DSS with bacterial broth on cell viability. **(C,D)** Relative quantitative RT-qPCR profiles of intercellular tight junction protein ZO-1 and occludin gene. **(E)** Western blot of intercellular tight junction proteins ZO-1 and occludin expression. **(F,G)** Quantitative Western blot of intercellular tight junction proteins ZO-1 and occludin. **(H,I)** ZO-1, occludin expression level was estimated by Immunofluorescence assay (*p < 0.05).

To study the protective effect of different fractions of *B. intestinihominis* against DSS-induced impairment of Caco-2 cell, CCK-8 cell viability assay performed firstly. Neither the fermentation broth, heat-inactivated *B. intestinihominis* solution, nor live bacteria exhibited cytotoxic effects on Caco-2 cells. Notably, the 10% fermentation broth significantly enhanced cellular viability (Figure 5A, Supplementary figure S1). 2.5% DSS was chosen to disrupt cell activity, and the results showed that 10% fermentation broth of the *B. intestinihominis* restored the cell viability in DSS-induced impairment cell model (Figure 5B). RT-qPCR detected the mRNA expression levels of tight junction proteins ZO-1 and occludin. mRNA expression levels of ZO-1 and occludin were significantly decreased in the DSS group compared with that of the NC group (*p* < 0.05), whereas the mRNA expression levels of ZO-1 were significantly increased (*p* < 0.05) after heat-inactivated *B. intestinihominis* solution (Figures 5C,D). The expression levels of tight junction proteins ZO-1 and occludin were detected by Western blotting, and the expression levels of ZO-1 and occludin were significantly decreased in the DSS group compared with the NC group (*p* < 0.05), the expression levels of ZO-1 increased significantly after intervention with heat-inactivated *B. intestinihominis* solution (*p* < 0.05). The fermentation broth of *B. intestinihominis*, heat-inactivated *B. intestinihominis* solution and live *B. intestinihominis* had no significant effect on the occludin protein expression (Figures 5E–G). Moreover, immunofluorescence indicated results that were consistent with our previous finding. Increased ZO-1 expression was found on the cell membrane, when heat-inactivated *B. intestinihominis* solution was used for intervention (Figure 5H). However, there was no significant change in the expression of occludin (Figure 5I). These findings suggest that heat-inactivated *B. intestinihominis* exerts specific protective effects on intestinal barrier function through upregulation of ZO-1 expression, while the fermentation broth enhances cellular viability.

### 3.6 *Barnesiella intestinihominis* has no significant effect on Caco-2 cell monolayer barrier permeability

**FIGURE 6:**
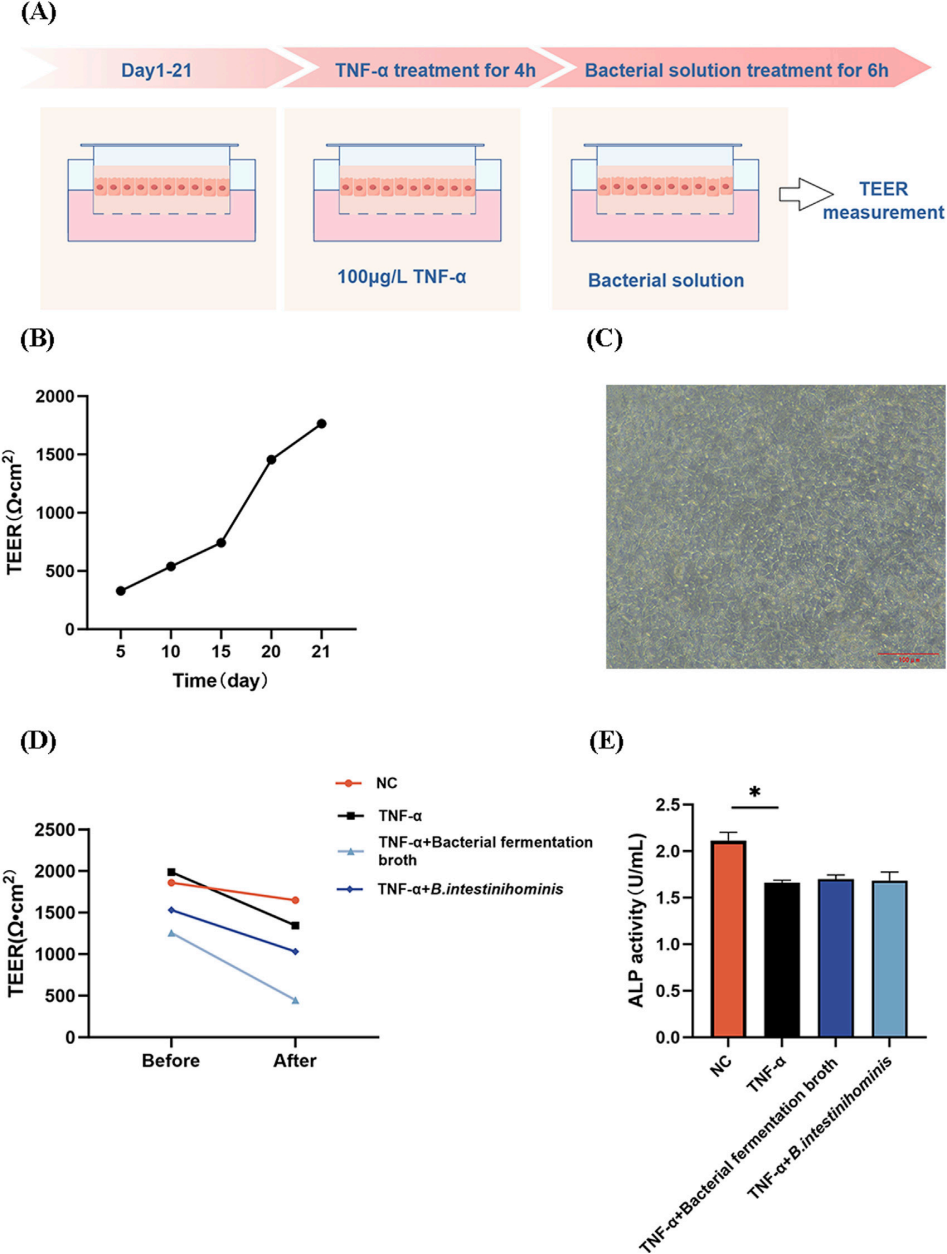
*Barnesiella intestinihominis* has no significant effect on Caco-2 cell monolayer barrier permeability. **(A)** Schematic diagram of the establishment of the Caco-2 monolayer cell barrier model. **(B)** TEER of Caco-2 cells with time. **(C)** Caco-2 cell morphology observed on day 21. Scale bar = 100 μm. **(D,E)** Changes in TEER and ALP activities before and after intervention (*p < 0.05).

To comprehensively assess the effect of *B. intestinihominis* on intestinal barrier function, we measured the trans-epithelial electrical resistance (TEER) and alkaline phosphatase (ALP) activity of Caco-2 monolayers (Figure 6A). TEER is an indicator of cellular barrier, and higher values represent better cellular barrier function. TEER values increased with incubation time during Caco-2 monolayer barrier establishment (Figure 6B). And as shown in Figure 6C, when the Caco-2 cells were cultured for 21 days, the cells were closely attached to each other without gaps. Compared with the NC group, the TNF-α-treated model group exhibited significantly reduced TEER values (*p* < 0.05). Notably, neither *B. intestinihominis* fermentation broth nor live *B. intestinihominis* suspension demonstrated significant TEER recovery compared to the model group (Figure 6C). Consistent with TEER findings, ALP activity analysis revealed marked reduction in the model group (*p* < 0.05) (Figure 6D).

## 4 Discussion

The abundance of *B. intestinihominis*, a short-chain fatty acid-producing intestinal anaerobic bacterium isolated from healthy populations, was significantly reduced in IGR and T2DM populations compared to healthy populations (Doi et al., 2024; Zhang et al., 2025). Research has reported that acetate produced by *B. intestinihominis* enhances FGF21 expression by inhibiting HDAC9 in liver cells and increasing H3K27ac modification, thereby improving metabolic disorders in diabetic mice (Zhang et al., 2025). In the present study, we observed a significant reduction in the relative abundance of *Ligilactobacillus* and *Barnesiella spp*. in the gut of mice in the IGR group, while the relative abundance of *Lachnoclostridium* was significantly elevated in comparison to the NC group. The intervention of *B. intestinihominis* resulted in alterations to the composition of the GM, an increase in the relative abundance of *Ligilactobacillus* spp. and a significant decrease in the relative abundance of *Lachnoclostridium* spp. It reversed the aforementioned changes in genus abundance and led to an improvement in the dysbiosis of the GM in IGR mice. As documented in the pertinent literature, *Ligilactobacillus* are lactic acid bacteria, and *Ligilactobacillus salivarius* are recognized as potential probiotics (Guerrero Sanchez et al., 2022). The present study revealed that *Ligilactobacillus* spp. were more prevalent in the NC group, whereas their relative abundance declined significantly in the HFD-induced IGR group. However, the abundance of *Ligilactobacillus* spp. increased following the administration of *Barnesiella spp*. (Liang et al., 2020). *Lachnoclostridium bouchesdurhonense*, a strain isolated from obese patients, has been identified as a potential colorectal cancer-associated bacterium (Amadou et al., 2016). This study observed that, compared with the NC group, the abundance of *B. intestinihominis* was significantly reduced in IGR mice. Following treatment with *B. intestinihominis*, although the abundance of *B. intestinihominis* itself did not significantly increase, the abundance of beneficial bacterial genera in the intestines of IGR mice was upregulated. Furthermore, this strain exhibited a negative correlation with pro-inflammatory factors IL-6, TNF-α, and 2h-PG, while showing a positive correlation with the anti-inflammatory factor IL-10. The metabolic products of *B. intestinihominis* are primarily acetate and succinate, which lower local intestinal pH and inhibit acid-sensitive pathogens; Additionally, butyrate is not only the primary energy source for colonic epithelial cells but also inhibits the expression of pathogenic virulence genes by suppressing histone deacetylases and promotes the differentiation of regulatory T cells (Tregs), thereby maintaining immune homeostasis. Therefore, *B. intestinihominis* primarily regulates changes in the intestinal microbiota structure through the production of short-chain fatty acids. In conclusion, these findings suggest that HFD-induced dysbiosis in IGR mice can be effectively ameliorated by *B. intestinihominis*, and that these microbial alterations may contribute to improved glucose homeostasis and reduced T2DM risk by enhancing intestinal barrier integrity, modulating mucosal immunity, and optimizing microbiota-host metabolic interactions.

The intestinal barrier represents a complex, multi-component system comprising intestinal epithelial cells, intercellular tight junctions, gut-associated immune cells, the mucus layer, and resident gut microbiota (GM) (Paone and Cani, 2020; Schoultz and Keita, 2020; Suriano et al., 2022). This intricate structure serves as a critical defense mechanism, maintaining intestinal homeostasis and preventing systemic dissemination of harmful substances. Compromised intestinal barrier interity has been increasingly implicated in the pathogenesis of type 2 diabetes mellitus (T2DM) (Régnier et al., 2021; Xue et al., 2019), with tight junction proteins zonula occludens-1 (ZO-1) and occludin playing pivotal roles in maintaining epithelial integrity (Kuo et al., 2022; Krug and Fromm, 2020). Furthermore, *Bifidobacterium bifidum* has been demonstrated to alleviate intestinal inflammation and enhance the expression of intestinal tight junction proteins (Li et al., 2024).

In the present study, we observed that the expression levels of intestinal tight junction proteins ZO-1 and occludin were diminished in the HFD-induced IGR group. Conversely, the expression levels of the two proteins were elevated in the *B. intestinihominis*-treated group following the administration of *B. intestinihominis*. It has been demonstrated that an imbalance in intestinal immune homeostasis is associated with an impairment in the intestinal barrier. TNF-α has been shown to downregulate the intestinal barrier-associated tight junction proteins ZO-1 and occludin, which consequently affects the intestinal barrier function (Ghosh et al., 2021). Impairment of the intestinal barrier results in increased intestinal permeability, facilitating the passage of harmful intestinal metabolites, such as LPS, into the host. LPS is a pro-inflammatory factor released from the cell wall of dead gram-negative cells in the intestine. This results in the triggering of inflammatory responses in the host, the further damage of the epithelial cells, an increase in intestinal permeability, and an increase in the ease with which harmful substances metabolised by bacteria in the intestinal tract enter the organism (Wu et al., 2019; Stephens and von der Weid, 2020). The present study demonstrated a significant reduction in circulating LPS levels in the serum of the organism following the intervention of *B. intestinihominis*. This indicates that *B. intestinihominis* was able to mitigate the inflammatory response and intestinal barrier permeability of the organism to a certain extent, and prevent LPS produced by intestinal bacteria from entering the bloodstream. T2DM is often accompanied by chronic low-grade inflammation and insulin resistance, which can be attributed to the blockage of insulin conduction, leading to the failure of insulin to function properly (Zhang et al., 2019), and insulin resistance is a consequence of impaired insulin conduction, which results in the malfunction of the insulin signaling pathway (Kahn et al., 2006). Some studies have demonstrated that in patients with T2DM, the levels of pro-inflammatory factors IL-6 and TNF-α are elevated (Jingjing et al., 2021). IL-6 has been shown to impede insulin receptor signalling pathway conduction by activating the STAT3-SOCS3 signalling pathway, whereas TNF-α has been demonstrated to inhibit tyrosine phosphorylation of insulin receptor substrate-1, which in turn affects its binding to insulin receptor and inhibits insulin signalling pathway conduction (Wang, 2016). Some studies have demonstrated a negative correlation between *Barnesiella spp*. and pro-inflammatory factors IL-6 and TNF-α (Wei et al., 2018). This study also revealed a similar correlation between *Barnesiella spp*. and these factors. A negative correlation was observed between *Barnesiella spp*. and colonic pro-inflammatory factors IL-6 and TNF-α, while a positive correlation was noted between *Barnesiella spp*. and colonic anti-inflammatory factor IL-10. Pro-inflammatory cytokines, such as IL-6 and TNF-α, are expressed in insulin target organs, including adipose tissue, liver and muscle, which affects insulin sensitivity (Ren et al., 2021). The present study revealed that the secretion levels of pro-inflammatory factors IL-6 and TNF-α and the secretion level of anti-inflammatory factor IL-10 were decreased in colon tissues and increased in the *B*. *intestinihominis*-treated group intervened by *B. intestinihominis*. This resulted in improved localised inflammation and glucose tolerance in the colonic tissues of the organism. This suggests that *B. intestinihominis* reduces the intestinal permeability of colonic tissues, blocks harmful substances in the intestinal tract outside the intestinal epithelial cells, reduces the entry of endotoxin and other harmful substances into the bloodstream, and has a protective effect on the intestinal barrier of IGR mice. These findings align with emerging evidence demonstrating the anti-inflammatory potential of *Barnesiella spp*. enrichment through various interventions, including prebiotic administration (Yao et al., 2021; Weiss et al., 2014) and plant extract supplementation (Nong et al., 2024; Tian et al., 2024).

Short-chain fatty acids (SCFAs) are metabolites produced by intestinal flora fermenting undigested complex carbohydrates in the gut, with acetic acid, propionic acid, and butyric acid being the most abundant, and are involved in important physiological functions such as digestion, immunity, and neurology in the human body, and are also related to the prevention and treatment of a variety of diseases including inflammatory bowel disease, obesity, T2DM, and cardiovascular disease (May and Den, 2023). SCFAs are important energetic substances for intestinal epithelial cells (IEC), promoting colon cell proliferation, enhancing intestinal barrier function, and inhibiting inflammatory responses (Zhang et al., 2023). Gallic acid significantly increases short-chain fatty acids (SCFAs) in feces by enhancing the abundance of beneficial bacteria that produce SCFAs in the gut. It activates G protein-coupled receptors (such as FFAR2 and FFAR3) and inhibits the TLR4/NF-κB signaling pathway, thereby reducing the production of pro-inflammatory cytokines and repairing the damaged intestinal barrier (Tian et al., 2025). In the present study *B. intestinihominis* fermentation broth restored the DSS-induced decrease in cell viability, but was not significant for the improvement of the intestinal barrier. On the contrary, the heat-inactivated bacterial solution increased the expression of the intercellular tight junction protein ZO-1, suggesting that the strain ameliorated the DSS-induced impairment of the cellular barrier. Probiotic inactivation bacterial solution is a high-temperature treatment that inactivates probiotics but preserves their bacterial structure. Inactivation ruptures the cell wall and releases cytoplasmic contents (bacterial lysates) such as DNA, cell wall components, peptidoglycan, lipophosphatidic acid, or heat-resistant bacterial hairs (Taddese et al., 2021). Studies have shown that heat-inactivated probiotics are equally useful in maintaining barrier integrity. For example, heat-inactivated *Lactobacillus rhamnosus* (strain OLL2838) has been shown to improve mucosal barrier permeability in mice with induced colitis (Miyauchi et al., 2009). In addition, heat-inactivated *Lactobacillus acidophilus* LB has also been shown to alleviate *E. coli*-induced increase in paracellular permeability of Caco-2 cells (Liévin-le Moal et al., 2007). Our findings with heat-inactivated *B. intestinihominis* extend these observations, suggesting that structural components of this bacterium contribute to intestinal barrier protection. This highlights the potential for developing novel therapeutic strategies utilizing non-viable probiotic formulations, which may offer advantages in terms of stability and safety while maintaining biological activity.

In conclusion, our findings demonstrate that *B. intestinihominis* intervention effectively ameliorates glucose intolerance and insulin resistance in IGR mice. These beneficial metabolic effects appear to be mediated through multiple mechanisms: (1) modulation of gut microbiota composition, characterized by increased relative abundance of beneficial *Ligilactobacillus* spp. and decreased levels of potentially pathogenic *Lachnoclostridium* spp.; (2) attenuation of local intestinal inflammation; and (3) enhancement of intestinal barrier function through upregulation of tight junction protein expression. The observed improvements in intestinal homeostasis and metabolic parameters suggest that *B. intestinihominis* exerts its protective effects through complex microbiota-host interactions, potentially involving microbial metabolite production, immune modulation by bacterial structure, and epithelial barrier reinforcement. However, the precise molecular mechanisms underlying *B. intestinihominis*-mediated improvements in glucose metabolism and intestinal barrier integrity remain to be fully elucidated and warrant further investigation through targeted metabolomic and mechanistic studies. Although the research have some limitations such as occludin expression were inconsistent *in vivo* and vitro experiment and lack of metabolomics analysis and intestinal permeability detection, but this discovery provides important clues for microecological intervention in metabolic diseases and has significant scientific value and potential application prospects.

## 5 Conclusion

Our results suggest that *B. intestinihominis* can effectively ameliorate high-fat diet induced IGR through multiple complementary mechanisms: (1) reducing inflammatory cell infiltration in colonic tissues, inhibiting pro-inflammatory cytokine production, and enhancing secretion of anti-inflammatory mediators; (2) attenuating localized inflammation in the colon, and improving the integrity of the intestinal barrier; and (3) regulating the composition of the intestinal microbiota. These multifaceted beneficial effects make *B. intestinihominis* a promising next-generation probiotic therapeutic agent for metabolic disorders and related gastrointestinal complications.

## Data Availability

The original contributions presented in the study are publicly available. This data can be found here: https://doi.org/10.6084/m9.figshare.30239689.v1.
