# Supplementary material for: Barnesiella intestinihominis improves gut microbiota disruption and intestinal barrier integrity in mice with impaired glucose regulation
Source: Front Pharmacol. 2025 Oct 8;16:1635579. doi: 10.3389/fphar.2025.1635579 (PMC12540160; doi:10.3389/fphar.2025.1635579)
Supplement: Supplementary file 1 [file DataSheet1.pdf]

Supplementary materials

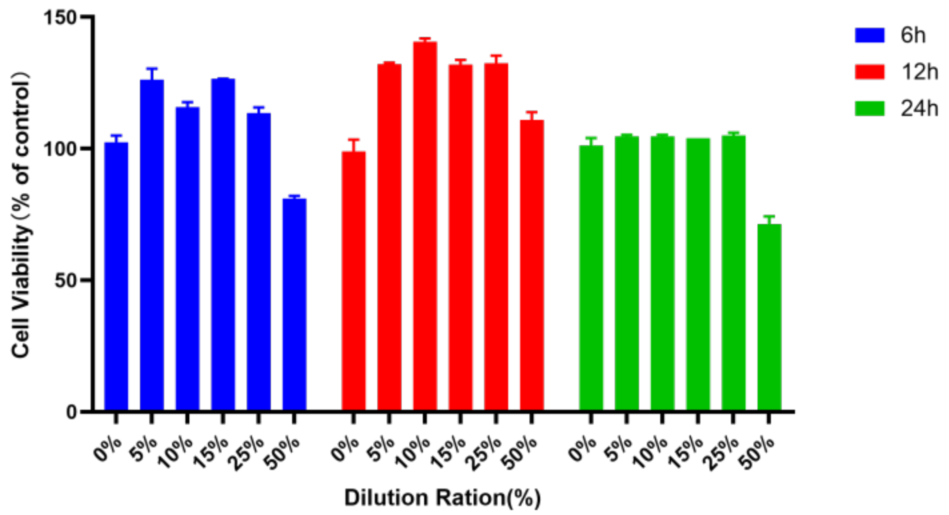

Fig. S1. Effect of different *B.intestinihominis* fermentation broth concentrations and intervention time on Caco-2 cell viability.

Gene primer sequences:

Gene name	Primer sequence (5'→3')
Mouse-β-Actin F	GGCTGTATTCCCCTCCATCG
Mouse-β-Actin R	CCAGTTGGTAACAATGCCATGT
Mouse-Occludin F	TTTCCTTAGGCGACAGCGG
Mouse-Occludin R	AAGATAAGCGAACCTGCCGAG
Mouse-ZO-1 F	GATGTTTATGCGGACGGTGG
Mouse-ZO-1 R	CATTGCTGTGCTCTTAGCGG
Human-β-Actin F	GAAGTCCCTTGCCATCCTAAA
Human-β-Actin R	GTCTCAAGTCAGTGTACAGGTAAG
Human-Occludin F	ACAAGCGGTTTTATCCAGAGTC
Human-Occludin R	GTCATCCACAGGCGAAGTTAAT
Human-ZO-1 F	CAACATACAGTGACGCTTCACA
Human-ZO-1 R	CACTATTGACGTTTCCCCACTC

Table S1. Gene primer sequences
